# Regulatory problems and developmental psychopathology within the first 2 years of living—a nested in cohort population-based study

**DOI:** 10.3389/frcha.2024.1330999

**Published:** 2024-02-13

**Authors:** Janni Ammitzbøll, Anne Lise Olsen, Susanne Landorph, Christian Ritz, Anne Mette Skovgaard

**Affiliations:** ^1^ National Institute of Public Health, Faculty of Health Sciences, University of Southern Denmark, Copenhagen, Denmark; ^2^ Center of Infant and Toddler Health, National Institute of Public Health, Copenhagen, Denmark; ^3^ Child and Adolescent Psychiatric Department, Region Sjaelland, Roskilde, Denmark

**Keywords:** regulatory problems (RP), infancy, developmental psychopathology, mental disorders, community-cohort

## Abstract

**Background:**

Infancy regulatory problems (RP) of sleep, feeding and eating, and excessive crying are thought to play a key role in the development of psychopathology in childhood, but knowledge of the early trajectories is limited.

**Objective:**

To explore RP at ages 8–11 months and the associations with mental health problems at 1½ years, and assess the influences of maternal mental health problems and relationship problems.

**Methods:**

RP was explored in a nested in-cohort sample (*N* = 416) drawn from a community-based cohort (*N* = 2,973). Cohort children were examined by community health nurses, using a mental health screening, which included seven items of RP. Follow-up at 1½ years included diagnostic assessment according to the International Classification of Diseases, ICD-10, and the Diagnostic Classification of Mental Health and Developmental Disorders in Infancy and Early Childhood: Revised edition, DC:0-3R. Data analyses included logistic regression models and analyses of the mediation effect of maternal mental health and relationship problems.

**Results:**

RP of sleep were associated with a 2-fold increased risk of child mental disorder specifically sleep disorders, adjusted odds ratio (OR) 9.3 [95% confidence interval (CI): 2.0–42.9], disorders of behavior and emotions, adjusted OR 2.9 (95% CI: 1.0–8.4), and DC:0-3R regulatory disorders, adjusted OR 2.7 (95% CI: 1.0–7.5). Children with RP of feeding and eating showed an increased risk of overall mental disorder, adjusted OR 1.4 (95% CI: 0.7–2.4), and specifically, feeding and eating disorders, adjusted OR 6.0 (95% CI: 1.6–21.7), disorders of behavior and emotions, adjusted OR 2.2 (95% CI: 0.9–5.8), as well as DC:0-3R regulatory disorders, adjusted OR 1.6 (1.0–7.5). RP of emotional regulation were associated with increased risk of any mental disorder, adjusted OR 1.5 (1.0–2.4), and specifically behavioral and emotional disorders, adjusted OR 2.2 (95% CI: 0.9–5.9) and DC:0-3R relationship disorders, adjusted OR 1.8 (95% CI: 0.9–3.8). The mediation effect of maternal mental health problems and relationship problems ranged between 0% and 48%.

**Conclusion:**

RP at ages 8–11 months is associated with increased risk of ICD-10 and DC:0-3R disorders at 1½ years. Study findings highlight a group of vulnerable infants in need of preventive intervention to break the early trajectories of psychopathology.

## Introduction

Mental health development in infancy and early childhood includes the dynamics of the infants' inborn capacities and the progressive integration of physiological and emotional influences, as well as the attentional and cognitive stimulation. Overall, the infants' development occurs within a frame of the increasingly differentiated reactions to internal and external sensory inputs, coregulated and nurtured by the parents (1, 2). Along with the maturation of child's self-regulation, the child becomes increasingly able to express a variety of needs and emotions that parents can respond to, and depending on the congenital resources of the infant and the resources of caregivers, this process can be facilitated or impaired (3). The regulation of sleep, appetite, feeding and eating, and sensitivity in reactions to internal as well as external stimuli are central features in the infants’ development, and studies of premature infants and infants with developmental vulnerabilities have shed light on the longitudinal impact of problems in the regulation of sleep, appetite, feeding and eating, and sensitivity in reactions to annoying internal and external stimuli (2, 4). Also, infants born at term and without developmental vulnerabilities may suffer from excessive crying, sleep problems or problems in feeding and eating. Nevertheless, these problems are mostly temporary, usually fading during the first 6 months of living, along with increasing maturity of the infants' regulatory capacities and the parents increasingly effective coregulation (2, 3). A minor part of the infant population may, however, suffer from persisting problems beyond 6 months, broadly conceptualized as temperamental difficulties or regulatory problems (RP). These problems challenge the parenting, and longitudinally they are considered to influence the mental health of the child (5). Within the field of early developmental psychopathology, problems on the regulation of neurophysiological and emotional/behavioral reactions have gained increasing interest in clinical settings (6), reflected by the conceptualization of regulatory disorders as a clinical entity (7) and the development of the Diagnostic Classification Zero-to-Three (8). The concept of RP—including excessive crying, sleep problems, and problems of feeding and eating (9–11) —have been investigated in clinical samples as well as community studies (9, 10), regarding both solitary problems of sleeping, crying, or feeding and eating, as well as co-occurring combined or complex RP. Longitudinal studies of RP cover a wide range of outcomes, including cognitive development (12, 13); attachment (14); attention (15); disorders of neurodevelopment, including pervasive developmental disorders and autism disorders (16–20) as well as attention deficit and hyperactivity disorder (ADHD) (11, 18, 19, 21–23). In particular, childhood behavioral problems and dysregulation (10, 11), and internalizing and externalizing problems (24) have been found to be associated with infancy RP. Notably, a dose–response pattern has been suggested in which increasing numbers of RP may track into developmental paths of dysregulation having onset in early childhood (11); and, possibly, showing persistence across development, leaving the child with an increased risk of symptoms of depression, and psychotic and borderline personality disorder in adolescence (25).

The risk factors of RP have repeatedly been shown to include a range of pre-, peri-, and postnatal factors associated with child neurodevelopmental vulnerability, including preterm birth, as well as factors associated with compromised psychosocial resources in the family (9, 10, 26). The importance of maternal mental health problems in early child development is increasingly documented (27–29), and the early parent–child relations and the quality of parenting is suggested to play a key role in the risk mechanisms leading to child psychopathology (18, 20). Specifically regarding RP, the developmental vulnerability of the child is suggested to be influenced by parents' mental health via parents' emotional availability, and—beyond nurturing and care—their ability to interpret and respond to the signals and reactions of the child (11, 15, 30–32). However, the available literature yields no firm conclusions regarding the influence of maternal mental health problems and parent–child relationship problems on the early risk mechanisms of RP and emotional and behavioral dysregulation and developmental psychopathology (9, 32, 33).

A meta-analysis including 22 clinical and community studies of infancy RP (10) found externalizing problems and problems of attention deficit and hyperactivity, ADHD, to be the strongest outcome of any regulatory problem, with a particularly high risk in families of multiple risks. This meta-analysis was recently updated to include a total of 30 clinical as well as community-based studies published in 1987–2020 (9). A total of 34,582 participants were included, assessed at baseline at 6.5 ± 4.5 months, and at follow-up at ages 5.5 ± 2.8 years. Most of the studies reviewed concerned excessive crying (13) and sleep (9), whereas feeding and eating problems were investigated in only three studies. The cumulative incidence for behavioral problems during childhood was 23.3% in children with RP, with a higher risk found among infants with multiple RP (9). The study limitations highlighted include the heterogeneity of the data and study samples, unvalidated definitions of RP, measurement problems, and reporting bias as most studies build on parents’ reports both at baseline and outcome. Moreover, the authors of the recent review underscore the need to include measures of parental health and the parent–child relation as potential mediators (9).

Longitudinal studies of combined regulatory problems in early childhood investigated in community-based studies have recently been specifically reviewed (32). This review highlights the gap in epidemiological studies of the early course of RP as advance markers of developmental psychopathology tracking into the range of mental health problems in early childhood (32).

In sum, the available research evidence converges on the longitudinal risk associated with RP in infancy, underscoring the need for research that specifically adds to the knowledge on early developmental trajectories of dysregulation as a prerogative to preventive intervention to address mental health in early childhood (9–11).

The possibilities of identification of infants in need of preventive intervention regarding mental health have previously been explored in the Copenhagen Child Cohort using the municipality child health surveillance and settings and routines of community health nurses (CHNs) as the frame (34). Based on the finding of a window of opportunity at child ages 8–11 months, a standardized measure, PUF [Mental Health Development (in Danish: Psykisk Udvikling og Funktion)], has been developed and validated to capture the range of potential mental health vulnerabilities in early ages. The PUF includes 28 items covering child development and behavioral and emotional regulation, of which three items concern sleep; four items feeding and eating; and four items emotional regulation, including excessive crying (16, 35, 36). The overall predictive validity of the PUF as a screening measure has previously been explored using an item–response analytic approach suggests that a cutoff at three or more problems across the 28 PUF items are predictive of psychopathology measured as ICD-10 diagnoses. In addition, previous investigation of the very early pathways of RP of sleeping, feeding and eating, and excessive crying measured as the CHNs global concern recorded at home visits between birth and 6 months, and RP measured using the PUF at 8–11 months (31), suggest that RP beyond the first 2 months of living may develop seemingly independent of early exposures to maternal mental health problems and parent–child relationship problems (31).

Nevertheless, research data on the developmental pathways of RP and associated psychopathology in the first years of living is scarce, leaving a gap in knowledge on targets of intervention to effectively address RP in a period of life in which intervention has the strongest preventive potential regarding the child's future mental health.

### Objectives

This study aims to extend the investigation of the longitudinal associations of RP measured in community settings at child ages 8–11 months and clinically diagnosed mental health problems and psychopathology at age 1½ years, with a specific focus on the mediating role of maternal mental health problems and mother–infant relationship problems in early infancy (0–6 months). We hypothesize that the different kinds of RP will be differentially associated with child psychopathology within the same area of mental health, but also that RP may act as a transdiagnostic marker of dysregulation and overall mental health vulnerability. Also, we hypothesize that the mediating effect of maternal mental health problems and mother–infant relationship problems on the risk associations may differ across areas of child psychopathology.

## Materials and methods

### Setting, study population, and design

The study was conducted within the healthcare settings of 11 Danish municipalities, in which CHNs offer home visits to all infant families. In these municipalities, the CHNs use a standardized record, at child ages 0–2, 2–6, and 8–11 months, to systematically register the information obtained regarding child health and development, parent–child relations, and the family background. The CHN's recordings include information from the parents as well as the results of the CHNs' child assessments and observations of parent–child relations at the home visit [for detailed information of the setting and records see Ref. (31)]. In the study municipalities, the CHNs use the PUF measure at the home visit at ages 8–11 months to make the overall evaluation of child mental health (35) (see Supplementary Appendix I).

The study population was consecutively enrolled from March 2011 to April 2013, and of 3,263 children enrolled, a total of 2,973 (91%) having full data on the PUF assessment at ages 8–11 months were included. A nested in-cohort sample of children with three or more problems according to the overall PUF assessments (*N* = 237), and children from a random sample of every seventh from the remaining cohort of low problems scores (*N* = 179) were diagnostically assessed at age 1½ years [see Ref. (16) for further description of the sampling]. The background characteristics of the study sample compared to the remaining cohort have been described in a previous paper (16), and are shown in Supplementary Appendix II.

### Measures

RP at 8–11 months were identified by CHNs using the PUF measure (16, 36), and we defined RP as at least one problem recorded in one of the domains of sleeping (three items), feeding and eating (four items), or emotional regulation including excessive crying (four items) (see Supplementary Appendix I). Combined RPs were defined as two or more simultaneously recorded RP in at least two domains of either sleeping, feeding and eating, and emotional regulation; all dichotomized into yes/no variables.

At 1½ years, the children were examined during a 2 hour session with the parent being present throughout, using a previously validated procedure (37, 38). The assessments were done by a child research nurse and an infant‒toddler psychologist. Assessments included interviewing the parents, developmental testing, as well as clinical observations and video recordings of the child's behavior during play and during a meal, both in interaction with the parents and in interaction with the examiners. The parents’ interview included the Mannheim Eltern Interview; the Child Behavior Checklist (CBCL) 1½‒5, parent version; the Checklist for Autism and Toddlers (CHAT) and the Infant Toddler Symptom Checklist (ITSCL) (for further description of the assessment measures see Ref. (38). An infant‒toddler psychologist examined the child's development using the Bayley Scales of Infant and Toddler Development (BSID II). Parent–child relations were examined using the Parent–Infant Early Relation Assessment, providing standardized information on the communication, interaction, and emotional relations between parent and child. In line with the procedure described in Skovgaard et al. (38), two child psychiatrists experienced in infant and toddler mental health (AO, AS) reviewed the information obtained at the child assessment to complete the diagnostic assessment in accordance with the defining criteria of the International Statistical Classification of Diseases, 10th Revision (ICD-10) (39) and the Diagnostic Classification of Mental Health and Developmental Disorders of Infancy and Early Childhood: Revised edition (DC:0–3R) (8). The diagnostic assessment included independent rating of all cases and discussion to obtain consensus in case of divergence among the two raters (38). The assessments were blinded to the group members of the high vs. the low score group. The reliability of diagnostic assessment at ages 1½ years using measures as in the present study has previously been established (37).

Danish population registries and the Community Health Nurses Record provided comprehensive information on a range of child and family variables, including child sex, gestational age, whether parents were cohabiting at the time of the child's birth, the parents' country of birth, and parents' years of schooling (35).

Assessments of maternal mental health problems and mother–child relationship in the first six postnatal months were done by CHNs at home visits at child ages 2–4 weeks, 2–3 months, and 4–6 months. During the home visits of mean 60 min, the CHN follows a standardized manual to evaluate whether the mother has mental health problems or not, based on the mother's information during the home visit, and the CHN's evaluation on the presence of depressive symptoms, anxiety, or other psychological problems (31). The CHN evaluates the parent–child relationship from the parents' description of their relation to the child and from her observation of the mother’s handling of the child, the emotional expressions, quality of contact, and the overall interaction between the mother and child. The CHN made her final recordings based on the presence of a problem or not, in accordance with the manual (31).

### Data analyses

Descriptive statistics included the frequencies of RP and the background characteristics of child and parents at baseline, and of ICD-10 and DC:0-3R disorders diagnosed at age 1½ years.

The items of RP were included in the analyses as dichotomized variables (Table 1). We analyzed outcome in the following main groups of ICD-10 diagnoses: neurodevelopmental disorders (F84, F88, F89, F90, F80.9, F80.2, F70.9, and R41.8); emotional and behavioral disorders (F91, F92, F93, F94.1, F94.2, and F94.8); feeding and eating disorders (F98.2); sleep disorders (F51); and the DC:0-3 R diagnoses regulatory disorders and relationship disorders. Associations between RP at age 8–11 months and the main groups of ICD-10 and DC:0–3R disorders were examined by logistic regression models and results were reported as odds ratio (OR) with 95% confidence interval (CI).

**Table 1:** Variables of regulatory problems (RP) assessed by community health nurses (CHNs) at child ages 8–11 months (*N* = 416). Stratified on children of high and low risk of mental health vulnerability.^a^

Regulatory problem	Description	Frequency, *n* (%)	
		High scores (*n* = 237)	Low scores (*n* = 179)
Sleeping			
Stable sleeping pattern Falling asleep time Interrupted sleep	The child has established a steady pattern for sleeping and being awake The child falls asleep within 1 hour The child is able to sleep at least three consecutive hours		
Any problem of sleep regulation		82 (34.5)	8 (4.5)
Regulation of eating and feeding			
Appetite regulation Eats too little Refusal to eat Vomiting without otherwise being ill	The child indicates clearly when it is hungry or full The child has to be pressured to eat enough The child refuses food even though it has not eaten for a long period of time The child vomits more than once a week		
Any problem of eating and feeding regulation		152 (63.9)	12 (6.7)
Emotional regulation, incl. excessive crying			
Generally happy and satisfied Often irritable, fussy, dissatisfied Cries often Emotionally blunted	The child is happy and satisfied more than 80% of its waking time The child has at least two episodes every day where it is irritable, fussy, dissatisfied The child cries more than 1 hour every day The child shows no happiness, has limited facial expression and seems sad more than 50% of its waking time		
Any problem of emotional regulation, incl. excessive crying		108 (45.5)	11 (6.2)
Combined problems			
Problems in ≥2 of the above areas	Any combination of problems from at least two of the above areas of sleeping, eating and feeding and emotional regulatory problems	112 (47.1)	- (-)^b^

^a^ Ammitzbøll et al. (16).

^b^ Less than five numbers in a cell are not reported to secure anonymity.

The regression models included four steps: first, we included sex, gestational age, cohabitating parent, parents' country of birth, and parent schooling. Second, we separately added maternal mental health. Third, we added mother–child relationship variables. Finally, we included all variables from steps 1–3 simultaneously. These analyses were based on complete-case data (omitted participants with any missing data for one or more of the variables adjusted for). Mediation analysis for binary outcomes was carried out following the general methodology by MacKinnon et al. (40). Specifically, proportions mediated for early maternal mental health problems and relationship problems were estimated by means of the ratio between the direct mediated effect and the total effect using estimates on the log odds scale (41). Accompanying approximate 95% confidence intervals were also estimated using the corresponding standard errors on the log odds scale. Due to the small sample size statistical tests were not applied as power was low as is the case for many mediation analyses (42). However, a tentative cutoff for claiming substantial mediation around 30%–40% may be used (41). Statistical analyses were performed with the statistical program SAS, version 9.4 (SAS Institute Inc., Cary, NC, USA).

## Results

The background characteristics of study participants (*N* = 416) are shown at Table 2, and for comparison with the entire cohort in Supplementary Appendix II.

**Table 2:** Background characteristics of the study participants (*N* = 416).

Sample characteristics	Frequency, *n* (%)	Missing, *n* (%)
Child factors		
Gender		
Male Female	213 (51.2) 203 (48.8)	0 (0)
Gestational age		
Preterm (<37 weeks) Term (≥37 weeks)	32 (7.9) 371 (92.1)	13 (3.1)
Family factors		
Parent born outside a Nordic country		
Yes No	108 (26.0) 308 (74.0)	0 (0)
Mother's years of schooling		
Low (≤10 years) High	28 (10.5) 239 (89.5)	149 (35.8)
Parents living together at child's birth		
No Yes	24 (6.0) 374 (94.0)	18 (4.3)
Mother mental health problems		
Yes No	117 (31.1) 259 (68.9)	40 (9.6)
Mother–child relationship problems		
Yes No	40 (10,6) 338 (89.4)	38 (9.1)

For a more detailed overview of the characteristics of the participants at 1½ years (*N* = 416) and the remaining cohort see Supplementary Appendix II.

The description and frequency of RP in the study sample are shown in Table 1. Overall, the most common problem at ages 8–11 months was RP of feeding and eating affecting 39.4% of the study participants, whereas RP of emotional regulation including excessive crying affected 28.6%, and RP of sleeping 21.6%. A total of 114 children (27%) had two or more RP.

Overall, a total of 115 children were diagnosed with an ICD-10 disorder, of which neurodevelopmental disorders and disorders of behavior and emotions, and adjustment disorders were the most common (see Supplementary Appendix III). Among DC:0-3R diagnoses, regulatory disorders were diagnosed in 38 children (9.1%); whereas the DC:0-3R diagnoses of relationship disorders were the most common of all diagnoses, found in 66 of the children (15.9%) (see Supplementary Appendix III).

The univariate associations of maternal mental health problems and mother–child relationship problems recorded between birth and child age 6 months, and RP at ages 8–11 months, and main groups of ICD-10 and DC:0-3R disorders at 1½ years are shown in Supplementary Appendix IVA and B. Univariate analyses showed no significant associations between early maternal and relationship problems and RP at 8–11 months, whereas maternal mental health problems were associated with increased risk of any ICD-10 disorder OR 1.7 (95% CI: 1.1–2.8) and DC:0-3R regulatory disorders OR 2.0 (95% CI: 1.0–4.1). Mother–child relationship problems were associated with increased risk of any ICD-10 disorder OR 2.1 (95% CI: 1.1–4.1), and in particular DC:0-3R relationship disorders OR 2.5 (95% CI: 1.2–5.2). Logistic regression models of the associations between RP and main groups of ICD-10 diagnoses at 1½ years are shown in Table 3. RP of sleep was associated with a more than 2-fold increased risk of any mental disorder, adjusted OR 2.6 (OR 95% CI: 1.3–5.2), and behavioral and emotional disorders, adjusted OR 2.9 (95% CI: 1.0–8.4). Associations were overall independent of adjustment for child perinatal and family adversities and maternal mental health problems and relationship problems in the first 6 months of the child's life. The mediation proportion of both maternal mental health problems and relationship problems were 11% (CI: 0–51) or less.

**Table 3:** Associations (OR) for main groups of ICD-10 of mental disorders at 1½ years by regulatory problems at 8–11 months and mediation proportion (MP %) of early (0–6 months) maternal mental health problems and relationship problems (*N* = 416).

	Any ICD-10 disorder^a^	Any ICD-10 disorder^b^	Any developmental disorder^a^	Any developmental disorder^b^	Any behavioral or emotional disorder^a^	Any behavioral or emotional disorder^b^
Regulatory problem	OR (CI)	OR (CI) MP^c^% (CI)	OR (CI)	OR (CI) MP^c^% (CI)	OR (CI)	OR (CI) MP^c^% (CI)
Sleeping problem (*n* = 90)	**2.9** **(1.8–4.7)**	**2.6** **(1.3–5.2)**	1.7 (0.8–3.6)	1.0 (0.3–3.3)	**2.2** (**1.1–4.7)**	**2.9 (1.1–8.2)**
		10% (0–45)		–		11% (0–51)
Eating and feeding problem (*n* = 164)	**1.5** **(1.0–2.4)**	1.3 (0.7–2.4) 22% (0–70)	1.0 (0.5–2.1)	0.5 (0.2–1.4) –	0.9 (0.4–1.8)	1.1 (0.4–3.1) –
Problems of emotional regulation incl. excessive crying (*n* = 119)	**1.5** **(1.0–2.4)**	1.1 (0.6–2.1) 48% (0–88)	1.1 (0.5–2.4)	0.5 (0.2–1.7) –	2.0 (0.9–4.0)	2.2 (0.9–5.9) 5% (0–56)
Combined RP (*n* = 114)	**2.3** **(1.4–3.6)**	**1.9** **(1.0–3.5)**	1.2 (0.5–2.5)	0.4 (0.1–1.4)	1.6 (0.8–3.3)	2.4 (0.9–6.4)
		7% (0–48)/ 13% (0–52)		–		4% (0–52)/ 8% (0–54)

Bold: *p*-value <0.05.

^a^ Unadjusted (*N* = 416).

^b^ Adjusted for gender, gestational age, parent born outside a Nordic country, mother less than 10 years of school, parents not living together at childbirth, and maternal mental health problems and mother–child relationship problems measured at child ages 0–6 months (*N* = 247).

^c^ MP: the mediation proportion % (CI) of early (0–6 months) maternal mental health and relationship problems by mean of the ratio between the mediated effect and the total effect.

RP of feeding and eating was associated with an increased risk of any ICD-10 disorder, however, attenuated in adjusted analyses, adjusted OR 1.4 (95% CI: 0.7–2.4). Maternal mental health problems in the first 6 months of the child's life accounted for a mediation proportion of 22% (CI: 0–70) on outcome, whereas relationship problems showed no influences. No significantly increased risk was found between RP of feeding and eating and behavioral and emotional disorders or neurodevelopmental disorders.

The associations of RP of emotional regulation including excessive crying and any ICD-10 disorder faded in analyses adjusting for a range of child, family, and relational adversities, adjusted OR 1.1 (95% CI: 0.6–2.1). The mediation proportion on outcome was highly influenced by relationship problems 48% (CI: 0–88), but not by maternal mental health (mediation proportion 0%). Regarding the associations between RP of emotional regulation and behavioral and emotional disorders, adjusted OR 2.2 (95% CI: 0.9–5.8), the mediating proportion of maternal mental health problems and relationship problems were 5% (CI: 0–50) or less. Combined RP was associated with increased risk of any ICD-10 mental disorder, adjusted OR 1.9 (95% CI: 1.0–3.5), and the mediating proportion of maternal mental health problems and relationship problems were 7% (CI: 0–48) and 13% (CI: 0–52), respectively. Likewise, an increased risk of behavioral and emotional disorders was found in children with combined RP, adjusted OR 2.4 (95% CI: 0.9–6.4), and the mediating proportion due to maternal mental health problems and relationship problems was 4% (CI: 0–52) and 8% (CI: 0–54), respectively.

Overall, no increased risk of neurodevelopmental disorders was found in children having infancy RP, whether being RP of sleeping, feeding, and eating, emotional regulation or combined RP.

As shown in Table 4, a pattern of association of problems within the same domain of mental health was seen in children with RP of sleeping, showing a more than 9-fold increased risk of an ICD-10 sleep disorder at 1½ years, adjusted OR 9.3 (95% CI: 2.1–42.9), mediated by less than 10% (CI: 0–42) by maternal mental health problems and relationship problems in infancy. Likewise, children with RP of feeding and eating showed a highly increased risk of feeding and eating disorder, adjusted OR 4.6 (95% CI: 0.9–22.7). Also, children with RP of emotional regulation showed an increased risk of behavioral and emotional disorders, adjusted OR 2.2 (95% CI: 0.9–5.8), overall indicating continuity of specific problems of dysregulation within the first years of living. Associations of problems from different areas of psychopathology were seen among children having RP of sleeping in infancy, being at an increased risk of feeding and eating disorders too, adjusted OR 3.7 (95% CI: 0.8–16.2), whereas no such patterns were seen regarding RP of feeding and eating, and RP of emotional regulation. The mediating proportion on outcome was 8% (CI: 0–48) or less.

**Table 4:** Associations (OR) for disorders of sleeping and eating and feeding at 1½ years by regulatory problems at 8–11 months and mediation proportion (MP %) of early (0–6 months) maternal mental health problems and relationship problems (*N* = 416).

	Eating and feeding disorder^a^	Eating and feeding disorder^b^	Sleep disorder^a^	Sleep disorder^b^
Regulatory problem	OR (CI)	OR (CI) MP^c^% (CI)	OR (CI)	OR (CI) MP^c^% (CI)
Sleeping problem (*n* = 90)	**2.8** (**1.0–8.4)**	3.7 (0.8–16.2)	**7.1** (**2.3–21.9)**	**9.3 (2.0–42.9)**
		–		8% (0–42)
Eating and feeding problem (*n* = 164)	**6.0** (**1.6–21.7)**	4.6 (0.9–23.7) –	2.1 (0.7–6.2)	2.4 (0.6–9.9) –
Problems of emotional regulation incl. excessive crying (*n* = 119)	0.4 (0.1–1.8)	0.8 (0.2–4.3) –	1.4 (0.5–4.3)	0.5 (0.1–3.0) –
Combined RP (*n* = 114)	**2.8** (**1.0–8.1)**	3.5 (0.8–14.5)	**5.1** (**1.7–15.5)**	**5.6 (1.2–25.6)**
		–		7% (0–48)

Bold: *p*-value <0.05.

^a^ Unadjusted (*N* = 416).

^b^ Adjusted for gender, gestational age, parent born outside a Nordic country, mother less than 10 years of school, parents not living together at childbirth, and maternal mental health problems and mother–child relationship problems measured at child ages 0–6 months (*N* = 247).

^c^ MP: the mediation proportion % (CI) of early (0–6 months) maternal mental health and relationship problems by mean of the ratio between the mediated effect and the total effect.

The outcome of DC:0-3R diagnoses of regulatory disorders (Table 5) was characterized by increased risk seen across the range of RP in infancy, but most pronounced in RP of sleeping, adjusted OR 2.7 (95% CI: 1.0–7.5). Overall, associations to DC:0-3R regulatory disorders faded in analyses adjusting for the range of child, family, and relational adversities (Table 5). Regarding sleeping RP, the mediating proportion of maternal mental health problems and relationship problems were 11% (CI: 0–52) and 18% (CI: 0–63), respectively, whereas no mediating effect was seen in children having RP of feeding and eating and emotional regulation including excessive crying.

**Table 5:** Associations (OR) for DC:0-3R diagnosis of regulatory and relationship disorders at 1½ years by regulatory problems at 8–11 months and mediation proportion (MP %) of early (0–6 months) maternal mental health problems and relationship problems (*N* = 416).

	Regulatory disorder^a^	Regulatory disorder^b^	Relationship disorder^a^	Relationship disorder^b^
Regulatory problem	OR (CI: 95%)	OR (CI) MP^c^% (CI)	OR (CI)	OR (CI) MP^c^% (CI)
Sleeping problem (*n* = 90)	**2.0 (1.0–4.2)**	**2.7 (1.0–7.5)**	1.5 (0.8–2.6)	1.4 (0.6–3.2)
		11% (0–52)/ 18% (0–56)		17% (0–72)
Eating and feeding problem (*n* = 164)	1.6 (0.8–3.1)	1.6 (0.6–4.2) –	1.2 (0.7–2.0)	1.4 (0.7–2.9) –
Problems of emotional regulation incl. excessive crying (*n* = 119)	1.7 (0.9–3.4)	1.2 (0.4–3.3) –	**1.8 (1.0–3.1)**	1.8 (0.9–3.8) –
Combined RP (*n* = 114)	1.4 (0.7–2.9)	1.5 (0.5–4.1)	1.4 (0.8–2.5)	1.7 (0.8–3.5)
		–		11% (0–63)

Bold: *p*-value <0.05.

^a^ Unadjusted (*N* = 416).

^b^ Adjusted for gender, gestational age, parent born outside a Nordic country, parents less than 10 years of school, patent living together at childbirth, and maternal mental health problems and mother–child relationship problems measured at 0–6 months (*N* = 247).

^c^ MP: the mediation proportion % (CI) of early (0–6 months) maternal mental health and relationship problems by mean of the ratio between the mediated effect and the total effect.

Associations of infancy RP and DC:0-3R relationship disorders were seen across the range of problems, but most pronounced for RP of emotional regulation including excessive crying, adjusted OR 1.8 (95% CI: 0.9–3.8). Notably, maternal mental health problems had no mediating effect on outcome, whereas mother–child relationship problems accounted for a mediating proportion of 17% (CI: 0–72).

Combined RP was associated with an increased risk of ICD-10 diagnosis, adjusted OR 1.9 (95% CI: 1.0–3.5), and particularly sleep disorders, adjusted OR 5.6 (95% CI: 1.2–25.6), whereas no significant associations were found to DC:0-3R regulatory and relationship disorders (Tables 3‒5).

## Discussion

In this longitudinal study, nested in a community-based cohort, we found infancy RP of sleeping, feeding and eating, and emotional regulation including excessive crying being associated with an increased risk of ICD-10 and DC:0-3R disorders diagnosed by experienced clinicians at child ages 1½ years. This increased risk persisted in analyses adjusted for exposures to a range of perinatal child and family adversities, including maternal mental health problems and relationship problems within the first 6 months of the child's life. Specific analyses focusing on the mediating proportion of maternal health problems and relationship problems showed less than 50% mediation.

The study was embedded in the municipality child healthcare in 11 municipalities in which the community health nurses, CHNs, use the validated PUF measure to screen for developmental vulnerabilities at child ages 8–11 months (35). The PUF measure includes validated items on RP of sleeping, feeding and eating, and emotional regulation including excessive crying (see Supplementary Appendix I). Most other studies of RP have been based on a diverse range of questionnaires to parents (9, 10, 32), leaving comparisons between studies challenging. Among studies using elaborated questionnaires, a study exploring RP over the first 18 months (33) in very preterm and preterm infants used items at 6 and 18 months which are very similar to ours, except for the feeding and eating item used in our study “vomits without otherwise being ill,” and three items on emotional regulation: “not happy and satisfied”; “often irritable and fussy”; “emotional blunted.” For comparison with the influential ALSPAC study (11), the same differences are seen regarding items concerning indicators of feeding and eating problems, whereas the indicators of sleeping problems overall correspond to the three sleep items used in our study at ages 8–11 months: “The child has not yet established a stable sleep pattern”; “difficulties falling asleep”; and “interrupted sleep.” The items on crying in the previously mentioned studies correspond to one of the items on emotional regulation in our study (16, 36), whereas our other indicators of emotional regulation are more in line with the one used in the Mannheim Study of Children at Risk, building temperamental difficulties (43).

The landmark study by DeGangi et al. (44) investigated symptoms of DC:0-3 regulatory disorders within the following domains: self-regulation, including variables on fussiness, irritability, crying, self-calming; attention; sleep; eating and feeding; tactile and auditory hypersensitivity; and emotional functioning using ITSCL, to parents (45). Based on a differentiated approach to the severity of RP in infancy and comprehensive measurements of child psychopathology at ages 30 months, this study highlighted that mild to moderate symptoms of regulatory disorders seem to fade over time, whereas severely disturbed children continued with impairing symptoms across the range of diagnoses investigated, including sleep disorders and parent–child relationship disorders (44). The measurements of regulatory problems in this study have much in common with the items used in our study, and the longitudinal findings do also concur with our findings, overall highlighting a group of regulatorily challenged children who suffer from persistent problems across their first years of living.

Findings from newer studies focusing on RP within the first years of living have shown a strong correlation of regulatory problems measured at ages from 6 to18 months (33) and up to 36 months (11). Among studies exploring mental health outcomes of infancy RP, several population-based cohorts have found an increased risk of parent-reported internalizing and externalizing or emotional and behavioral problems and disorders in older ages (11, 25, 30, 46, 47). In line with previous findings on mental health outcomes in older children, we found a highly increased risk of behavioral and emotional disorders identified already at 1½ years. Further, by including the range of child mental health problems and disorders in a comprehensive examination at follow-up, we extend the current understanding of the early pathways of dysregulation and developmental psychopathology in young ages (33, 44). We thus found homotypic stability of problems within the same domain e.g., infancy problems of sleeping being associated with clinical symptoms and diagnoses of sleep disorders at 1½ years; and feeding and eating problems being associated with symptoms and diagnoses of feeding and eating disorders at 1½ years. Also, a tendency of heterotypic association was seen among children with RP of sleeping and ICD-10 disorders of feeding and eating, overall indicating a transdiagnostic impact of having any RP. Homotypic continuity is characterized by consistency, similarity, and predictability of behaviors or internal states across different developmental phases, whereas heterotypic continuity does not imply longitudinal consistency of specific problems or symptoms, but rather an unspecific predictability across domains of mental health (48). Our findings based on data from very early childhood thus add to the knowledge on homotypic and heterotypic stability of psychopathology, which hitherto mostly have been explored in older ages.

Analyses of the mediating proportion of maternal mental health problems and mother–child relationship problems in early infancy indicate that maternal health problems may have an impact on the outcome in infants having RP of feeding and eating, whereas parent–child relationship problems seem to influence the outcome in infants with RP of emotional regulation in particular. In contrast, problems with sleeping appeared to be influenced to a lesser extent in general, but equally by maternal mental health problems and mother–child relationship problems. Still, our findings are far from conclusive due to small sample size and low power of calculations. However, being based on a community-based population and including the adjustment for a range of potential confounders, the study findings add to the current knowledge on the complex longitudinal influences of parental and relational factors (18, 49, 50). Previous research has highlighted the mediating role of maternal depression and anxiety and the impact of parental sensitivity on infants' stress reactivity and dysregulation (24, 30, 51–54), underscoring the overall importance of further investigation of the link between infancy RP and parenting quality (15). As suggested, this approach may run in parallel with research in methods to improve parenting among regulatory vulnerable infants (55, 56).

Overall, the findings from the present study agree with findings from previous longitudinal population-based studies exploring outcomes of infancy RP (9, 10). Of these, the vast majority have been studying parent-reported internalizing and externalizing problems and disorders in school-ages and beyond [see Ref. (32) for an overview]; a few have investigated neurodevelopmental disorders in clinically referred children (19, 22). Across these studies exploring a longer developmental period, the risk of unmeasured confounding is considerable, overall hampering the possibilities of identification of overt targets of preventive intervention in early childhood (32).

In contrast, we have been able to describe risk associations and developmental psychopathology within a narrow age span and in a developmental period, in which intervention has the highest preventive potentials. Moreover, by describing frequently occurring developmental phenotypes of impairing child problems that challenge parenting in a key developmental period, our study findings may be directly transferable to clinical and public health settings, in which parents of infants with RP have contact with health professionals, and so potentially ameliorate the possibilities of early identification and timely intervention.

Early identification of developmental vulnerability has to run in parallel with strategies to promote child development and reduce the risk of mental health problems and disorders (57), optimally integrating sensitive parenting and social learning as described, for example, in the Video-based Intervention to Promote sensitive Parenting (VIPP), which has shown promising results, also among regulatory disturbed infants (56, 58, 59).

### Strengths and limitations

The major strengths of the study include the large population-based study sample; the standardized assessments of RP at baseline (35); information on RP; and child, relational and family characteristics from standardized municipality records, as well as data on possible confounders obtained from national registries. Further strengths include the comprehensive face-to-face examinations at 1½ years and the diagnostic classification of psychopathology according to ICD-10 and the Diagnostic Classification of Mental Health and Developmental Disorders of Infancy and Early Childhood, DC:0-3R (8) with established reliability (37).

Notably, the combination of information from parents and community health nurses at baseline, and the follow-up assessments blinded of RP at baseline, by experienced clinicians, altogether optimizes the validity of data collected, and overall reduces the risk of information bias.

The study is embedded in existing service settings and carried out by CHNs which is a strength regarding the generalizability of the results of the study.

However, the strengths of being based on existing municipality settings and the existing routines of the CHNs must be understood in relation to the psychometric uncertainties given by the conditions of measuring RP within these settings. The items included as measures of RP in our study have been preliminary examined concerning the construct and predictive validity (16, 36), but the reliability of the CHNs assessments and recordings of the RP items has not been established.

Notably, the most important limitations of the study concern the validity of the concept of RP, being far from well-defined, leaving considerable methodological challenges to the assessments and classification of RP, and thereby challenging comparisons between studies. Still, these limitations are universal to all studies of RP, as highlighted in recent reviews (9, 32).

Another limitation of our study concerns the potential selection bias, given a lower participation of children from families of higher socioeconomic risks, but on the other hand, a relatively higher participation of children having maternal mental health and relationship problems reported within the first 6 months of the child's life. This selective dropout has been accounted for in the adjusted analyses, and overall, it is not considered to qualitatively influence the results found (60).

Even though our sample size was not very small (*N* = 416), several of the variables recorded in the municipality health nurse record were missing. This particularly influences the power of mediation analyses, which therefore mostly should be seen as data exploration for generating hypotheses about potential mediation effects. Notably, as missing data appeared to occur completely at random, our findings based on complete-case (adjusted) analyses remain unbiased.

## Conclusion, implications, and future research

Children who show RP of sleeping, feeding and eating, and of emotional regulation including excessive crying in late infancy (8–11 months) have an up to doubled risk of mental disorders at 1½ years, most pronounced regarding RP of sleeping. Exploring a population-based sample and being able to adjust for potential perinatal or psychosocial family determinants, the study findings point to RP as important markers of mental health vulnerabilities that should be addressed promptly to prevent the progression from dysregulation tracking into child psychopathology in older ages.

The phenomenology and developmental pathways of RP shown call for methods of preventive intervention that focus on parents’ abilities to understand and meet their child's regulatory vulnerability in a sensitive and developmentally appropriate way. These methods should be applied in clinical as well as in public health settings, such as the municipality child healthcare service; and overall, be built on evidence-based measures to promote sensitive parenting and strategies of social learning.

However, more research is needed to validate the clinical and developmentally important aspects of regulatory problems in early childhood, as well as research into measures of the reliable identification of these problems. Likewise, more research is needed to qualify effective and feasible methods of sensitive parenting with the potential to break the developmental trajectories of dysregulation and psychopathology early in childhood.

## Data availability statement

The original contributions presented in the study are included in the article/Supplementary Material, further inquiries can be directed to the corresponding authors.

## Ethics statement

The study was conducted as part of existing services in the participating municipalities. The Research Ethics Committee of the Capital Region of Denmark was conducted and had no demands on further clearance. The Danish Data Protecting Agency accepted the project as a subproject in the notification of the CHD, J.nr. 2010-54-1044. Parents gave their consent for participation in the PUF assessment at age 9–10 months and at age 1½ years.
