# Supplementary material for: Regulatory problems and developmental psychopathology within the first 2 years of living—a nested in cohort population-based study
Source: Front Child Adolesc Psychiatry. 2024 Feb 13;3:1330999. doi: 10.3389/frcha.2024.1330999 (PMC11748898; doi:10.3389/frcha.2024.1330999)
Supplement: Supplementary file 1 [file Datasheet1.pdf]

SUPPLEMENTARY MATERIALS

Appendix I. Overview of the PUF measure by domains, items and items description

Domain	Item	Description		
Sleep regulation	Stable sleeping pattern	The child has established a steady pattern for sleeping and being awake	Yes	no
	Falling asleep time	The child falls asleep within one hour	Yes	no
	Interrupted sleep	The child is able to sleep at least three consecutive hours	Yes	no
Eating	Appetite regulation	The child indicates clearly when it is hungry or full	Yes	no
	Eats too little	The child has to be pressured to eat enough	Yes	no
	Refusal to eat	The child refuses food even though it has not eaten for a long time	Yes	no
	Vomiting without otherwise being ill	The child vomits more than once a week	Yes	no
Expression of emotions	Generally happy and satisfied	The child is happy and satisfied more than 80% of its waking time	Yes	no
	Often irritable, fussy, dissatisfied	The child has at least two episodes every day where it is irritable, fussy, dissatisfied	Yes	no
	Cries often	The child cries more than one hour every day	Yes	no
	Emotionally blunted	The child shows no happiness, has limited facial expression and seems sad more than 50% of its waking time	Yes	no
Curiosity and interest	Curiosity, exploring	The child shows interest in its surroundings, examines its toys	Yes	no
Attention	Is able to focus	The child watch something or listen for more than one minute	Yes	no
	Maintain concentration	The child is able to examine toys for more than two minutes	Yes	no
	Easily distracted	The child is distracted by sounds, lights, movements, even while playing and does not return to its original activity	Yes	no
Motor activity	Generally increased level of activity	The child is characterized by a high level of activity restlessness	Yes	no
	Generally reduced level of activity	The child has a passive motoric, is mainly inactive	Yes	no
	Impulsiveness	The child is unpredictably active, throws things suddenly	Yes	no
Social communication and interaction	Eye contact	The child is able to establish eye contact. The Visiting Nurse is not in doubt that the child sees her eyes	Yes	no
	Contact smile	The child smiles to the Visiting Nurse when eye contact is made	Yes	no
	Proximity seeking	The child seeks contact with smiling, chattering, touching or reaching out after its parents	Yes	no
	Mutual communication	The child uses gestures, smiles and chatter with its parents for more than two communication loops (answer><reply)	Yes	no
	Joint attention	The child pays attention to parents' indications, checks and looks again	Yes	no
	Bodily contact	The child shows interest in bodily contact by expression and gesture	Yes	no
	Selectivity	The child clearly prefers the familiar care-personnel	Yes	no
Language	Language understanding	The child reacts to gestures/and some words	Yes	no
	Verbal expression	The child expresses itself with facial expressions, gestures, pointing, chatter in syllables	Yes	no

Ammitzbøll, J., Holstein, B.E., Wilms, L., Andersen, A. and Skovgaard, A.M. (2016) A new measure for infant mental health screening: development and initial validation. BMC Pediatr, 16(1), 197. https://doi.org/10.1186/s12887-016-0744-1

Appendix II. The background characteristics of the overall cohort (3,253) compared to the study population in this study (N=416).

Characteristics of participants at 1½ years (N=416) and the rest of study population (N=3253)

Variable	Participants at 1½ years (n=416) % (n)	Rest of the population (n=2837) % (n)	Participants versus non-participants p-value (missing n)
Child factors			
Child sex			
Boys	51.2 (213)	51.8 (1469)	.83 (0)
Girls	48.8 (203)	48.2 (1368)	
Gestational age			
Term	92.1 (371)	94.6 (2563)	.04 (141)
Preterm born (<37 weeks)	7.9 (32)	5.4 (146)	
Birth weight			
High birth weight	94.9 (388)	95.5 (2606)	.60 (114)
Low birth weight (<2500 grams)	5.1 (21)	4.5 (124)	
Neonatal complications			
No	77.2 (321)	78.9 (2237)	.43 (695)
Yes	22.8 (95)	21.1 (600)	
Apgar score			
High	92.9 (379)	94.5 (2572)	.19 (124)
Low (<10 at 5 min)	7.1 (29)	5.5 (149)	
Parental factors			
Mother smoking in pregnancy			
No	87.7 (357)	87.5 (2375)	.91 (132)
Yes	12.3 (50)	12.5 (339)	
Mother young at child birth			
No	93.0 (384)	91.2 (2512)	.23 (86)
Yes (<24 years)	7.0 (29)	8.8 (242)	
Father young at child birth			
No	98.3 (399)	95.9 (2596)	.02 (140)
Yes (<24 years)	1.7 (7)	4.1 (111)	
Mother born outside of Scandinavia			
No	82.2 (342)	78.3 (2220)	.07 (0)
Yes	17.8 (74)	21.7 (617)	
Father born outside of Scandinavia			
No	81.5 (339)	77.0 (2184)	.04 (0)
Yes	18.5 (77)	23.0 (653)	
Parents living together at child birth			
Yes	94.0 (374)	92.4 (2460)	.26 (192)
No	6.0 (24)	7.6 (203)	
Mother's years of schooling			
High	89.5 (239)	83.7 (1400)	.02 (1314)
Low (≤ 10 years)	10.5 (28)	16.3 (272)	
Father's years of schooling			
High	91.6 (186)	86.6 (1083)	.05 (1800)
Low (≤ 10 years)	8.4 (17)	13.4 (167)	
Mother mental health problems			
No	68.9 (259)	73.8 (1753)	.04 (503)
Yes	31.1 (117)	26.2 (621)	
Mother-child relationship			
No	89.4 (338)	91.1 (2168)	.28 (496)
Yes	10.6 (40)	8.9 (338)	

Ammitzbøll, J., Thygesen, L.C., Holstein, B.E., Andersen, A., and Skovgaard, A.M. (2018). Predictive validity of a service-setting-based measure to identify infancy mental health problems: a population-based cohort study. Eur Child Adolesc Psychiatry, 27(6), 711-723. https://doi.org/10.1007/s00787-017-1069-9

Appendix III: ICD-10 and DC:0-3 disorders diagnosed at age 1½ years.

ICD-10 and DC:0-3R disorders diagnosed at age 1½ years (N=416)

**ICD-10 diagnoses**	**Frequency** n (%)	**DC:0-3R child diagnoses**	**Frequency** n (%)
Any neuro-developmental disorder	33 (7.9)		
Any behavioral/emotional disorder	33 (7.9)	Regulatory disorders	38 (9.1)
Disorder of feeding and eating	14 (3.4)		
Sleep disorders	14 (3.4)		
		Relationship disorders	66 (15.9)
Other diagnosis	26 (6.3)		
Children with any ICD-10 diagnoses (one or more ICD-10 diagnoses)	115[1] (27.6)		

[1]Five children with more than one ICD-10 diagnose.

Appendix IV: The univariate associations of maternal mental health and relationsship problems and regulatory problems (RP) at ages 8-11 months and ICD10/DC:0-3 disorders at 1½ years.

a. The associations of maternal mental health problems and mother-child relationship problems at ages 0-6 months and regulatory problems (RP) at ages 8-11 months, OR (95% CI) (N = 416).

	Sleeping problems OR (CI 95%)	Feeding problems OR (CI 95%)	Crying problems OR (CI 95%)	Combined problems OR (CI 95%)
Mother mental health problems (0-6 months)	1.3 (0.7-2.2)	0.9 (0.6-1.4)	1.2 (0.8-2.0)	0.9 (0.6-1.5)
Mother-child relationship problems (0-6 months)	1.4 (0.7-3.1)	1.0 (0.5-1.9)	1.4 (0.7-2.8)	1.2 (0.6-2.5)

b. The associations of maternal mental health problems and mother-child relationship problems at ages 0-6 months and ICD-10 and DC:0-3 disorders diagnosed at age 1½ years, OR (95% CI) (N = 416)

	Any ICD-10 disorder OR (CI 95%)	Any developmental disorder OR (CI 95%)	Any behavioural disorder OR (CI 95%)	Eating and feeding disorder OR (CI 95%)	Sleep disorder OR (CI 95%)	Adjustment disorder OR (CI 95%)	Regulatory disorder OR (CI 95%)	Relationship disorder OR (CI 95%)
Mother mental health problems (0-6 months)	**1.7 (1.1-2.8)**	1.7 (0.8-3.8)	1.3 (0.6-2.9)	0.6 (0.2-2.2)	2.3 (0.8-6.7)	2.0 (0.9-4.5)	**2.0 (1.0-4.1)**	1.4 (0.8-2.5)
Mother-child relationship problems (0-6 months)	**2.1 (1.1-4.1)**	1.5 (0.5-4.4)	1.3 (0.4-4.0)	1.4 (0.3-6.6)	5.2 (1.7-16.5)	1.8 (0.6-5.5)	**2.4 (1.0-5.8)**	**2.5 (1.2-5.2)**
